# Fruit and Vegetable Intake and All-Cause Mortality in a Chinese Population: The China Health and Nutrition Survey

**DOI:** 10.3390/ijerph18010342

**Published:** 2021-01-05

**Authors:** Yuxuan Gu, Yansu He, Shahmir H. Ali, Kaitlyn Harper, Hengjin Dong, Joel Gittelsohn

**Affiliations:** 1 Department of Social Medicine, School of Public Health, Zhejiang University School of Medicine, 866 Yuhangtang Road, Hangzhou 310058, China; guyuxuan@zju.edu.cn; 2 The Jockey Club School of Public Health and Primary Care, Faculty of Medicine, The Chinese University of Hong Kong, Hong Kong, China; 1155139267@link.cuhk.edu.hk; 3 Department of Social and Behavioral Sciences, School of Global Public Health, New York University, New York, NY 11201, USA; sha371@nyu.edu; 4 Center for Human Nutrition, Department of International Health, Johns Hopkins Bloomberg School of Public Health, Baltimore, MD 21205, USA; kharpe14@jhmi.edu (K.H.); jgittel1@jhu.edu (J.G.)

**Keywords:** fruit, vegetables, mortality, prospective studies, China

## Abstract

This study was to investigate the association of long-term fruit and vegetable (FV) intake with all-cause mortality. We utilized data from the China Health and Nutrition Survey (CHNS), a prospective cohort study conducted in China. The sample population included 19,542 adult respondents with complete mortality data up to 31 December 2011. Cumulative FV intake was assessed by 3 day 24 h dietary recalls. Cox proportional hazards regression was used to estimate hazard ratios (HRs) and 95% confidence intervals (CIs) of all-cause mortality. Covariates included sociodemographic characteristics, lifestyle factors, health-related factors, and urban index. A total of 1409 deaths were observed during follow-up (median: 14 years). In the fully adjusted model, vegetable intake of the fourth quintile (327~408 g/day) had the greatest negative association with death compared to the lowest quintile (HR = 0.63, 95% CI: 0.53–0.76). Fruit intake of the fifth quintile (more than 126 g/day) had the highest negative association (HR = 0.24, 95% CI: 0.15–0.40) and increasing general FV intake were also negatively associated with all-cause mortality which demonstrated the greatest negative association in the amount of fourth quintile (HR = 0.59, 95% CI: 0.49–0.70) compared to the lowest quintile. To conclude, greater FV intake is associated with a reduced risk of total mortality for Chinese adults. High intake of fruit has a stronger negative association with mortality than differences in intake of vegetables. Our findings support recommendations to increase the intake of FV to promote overall longevity.

## 1. Introduction

Fruit and vegetable (FV) intake has been recognized as an important component of a healthy diet and has become a key component of multiple national dietary guidelines, including those in China [1,2]. Currently, the intake of vegetables by residents in China is gradually decreasing, and the intake of fruits remains low [3]. Chinese dietary guidelines recommend that vegetables consumption should be 300~500 g/day, and that fruits consumption should be 200~350 g/day [4]. However, according to the results of a sample of residents from 2010 to 2012, the average daily intake of vegetables and fruits for urban and rural residents in China is 269.7 g and 40.7 g, respectively [5]. Only half of the Chinese population meet the criteria of fruit and vegetable intake recommended by the Chinese dietary guidelines [6].

Past research on the relationship between food and human health has observed inadequate intake of fruits and vegetables to be among the top ten risk factors of death around the world [7]. Vegetables and fruits are rich in vitamins, minerals, and dietary fiber, and low in fat, salt, and sugar. They play an important role in meeting the human needs for micronutrients, maintaining the normal function of the human intestines, and reducing the risk of chronic diseases. Fruits and vegetables also contain various plant compounds, organic acids, aromatic substances, and pigments, which can increase appetite, help digestion, and promote overall human health [8].

Multiple studies have also shown that FV intake is closely and inversely associated with mortality. A nationwide Chinese study sampling more than 0.5 million adults aged 30–79 years concluded that fruit intake was associated with significantly lower mortality from several major vascular and non-vascular diseases [9]. This point was also strengthened by a systematic review including 142 publications, concluding that FV intake was associated with reduced risk of cardiovascular diseases, cancer, and all-cause mortality [10]. In Asian countries, the types of fruits and vegetables eaten or cooking methods may be different, hence this different FV profile might result in a disparate impact on health. In China, evidence to demonstrate that higher FV intake may be associated with significantly lower mortality from major cardiovascular disease and total mortality is limited [9,11,12]. However, some mixed results and inconsistencies remain. Conversely, a recent study by Wang et al. found no confirmed association between FV intake and mortality [13]. Until now, few studies have looked at a nationwide Chinese sample and investigated the association between both separate and combined fruit and vegetable intake and mortality. This study aimed to examine the association of combined FV intakes and separate fruit and vegetable intake with all-cause mortality utilizing data from a nationwide longitudinal sample.

## 2. Methods

### 2.1. Data Source

Data were drawn from the China Health and Nutrition Survey (CHNS) over a 20-year period, which is a prospective open cohort sample across nine provinces in China. Further details on the study design are described elsewhere [14]. The CHNS began enrolling individuals in 1989 and completed the ninth wave of data collection in 2011. Multistage random cluster sampling was used to sample the provinces. Questionnaires and anthropometric data were collected in 1989, 1991, 1993, 1997, 2000, 2004, 2006, 2009, and 2011. The CHNS study protocol and data collection procedures were approved by Institutional Review Boards of the University of North Carolina, Chapel Hill, and the Chinese Centre for Disease Control. All participants provided written informed consent. Self-reported questionnaire data included information on sociodemographic characteristics, lifestyle factors, body weight and body height, disease history, and urbanicity. Adults aged ≥ 18 years with complete data on age, sex, dietary, mortality information, and covariates were included in the present analysis. Pregnant women were excluded due to abnormal BMI. According to a previous study [15], participants with cardiovascular disease (CVD) (defined as a self-reported history of heart disease, stroke or hypertension) or cancer (defined as a self- reported history of cancer) at baseline were excluded, because these diseases would increase mortality in short-term which might shift the association between FV and mortality to null associations. The final analytical sample included a combined 19,542 participants with complete data of covariates.

### 2.2. Measurement

#### 2.2.1. Exposure

FV intake were assessed by a 3 day 24-h recall (two weekdays and one weekend) to record the amount (grams) each participant consumed on average in a 24-h period as well as the household level in the same three-day period. Individual intake data were collected by asking each family member separately to report all of the food that they had consumed at home and away from home with the 3 day, 24-h recall method. Household dietary intake data were calculated by examining changes in the inventory from the beginning to the end of the survey which used a combination of weighing and measuring techniques. The amount of food in each dish and the proportion of each dish which was consumed were noted by interviewers. During the questioning process, vivid pictures and food models were used by trained interviewers to help participants to recall the types, amounts, and places of each meal they consumed in the previous day. Two elements helped to control the quality of data collected. First of all, all interviewers were trained systematically with a high-quality course for more than three days. Secondly, interviewers compared the difference of food intake on an individual level and the individual’s average intake calculated from the household inventory. If significant differences existed, interviewers revisited the individuals and their corresponding families together, and collected the information of individual food intake. The intakes of FV were calculated by combining the amounts from each food category using corresponding editions of the Chinese Food Composition Table [16]. There were 3 versions used in the survey; the 1991 version was used for the dietary information before 2000, and the 2002 and 2004 versions were utilized for the 2004 and later rounds. The cumulative means of FV intake were calculated from all available rounds to represent the long-term diet and minimize individual variation. The complete dietary information was calculated as the average of up to 9 (average presumably approximately 4) separate 3-day diaries collected every 2–4 years. However, if some participants had fewer dietary records, then the dietary information was represented by that available data. FV intake was categorized into five quintiles. However, for fruit intake, it was a fact that in China almost half of the population did not consume fruit. So, we categorized the intake of 0 into the first quintile group, and then the remaining participants were organized into 4 smaller groups.

#### 2.2.2. Outcome

All-cause mortality was derived from the CHNS study. Follow-up periods were calculated from baseline until death or censoring (left the study before 2011 for another reason other than dying) on 31 December 2011, whichever was earlier. In CHNS, mortality status was ascertained by household information collected in each wave. If death was reported more than once, then the first reported date was used. Death from specific causes (illness, old age, accident or others) was only reported in the 1991 survey and there was no information on the cause of death from specific diseases in other waves of CHNS.

#### 2.2.3. Covariates

Potential covariates included sociodemographic variables, lifestyle behavior variables, and health-related variables. All covariates we used were from baseline data. Sociodemographic variables included age, sex, the highest level of education (graduated from primary school, lower middle school degree, upper middle school degree, technical or vocational degree, university or college degree, master’s degree or higher, or unknown), family income, marital status (never married, married, divorced, widowed, separated or unknown), and residence (urban, rural). Lifestyle behavior variables included smoking status, alcohol drinking, physical activity, and total calorie intake. Health-related variables included body mass index (BMI) (derived from measured/self-reported height and weight; categorized as underweight [<18.5 kg/m^2^], normal weight [18.5–23.9 kg/m^2^], overweight [24–27.9 kg/m^2^], or obese [≥28 kg/m^2^]), and disease history (cancer, hypertension, heart disease, and stroke). Height was measured to the nearest 0.2 cm using a portable stadiometer and weight was measured to the nearest 0.1 kg using a calibrated beam scale; BMI was calculated as weight (kg) divided by height squared (m^2^). We also included an urban index which reflects urbanicity and was calculated at the community level for each survey year using a multicomponent continuous scale. Communities could receive a maximum of 10 points for each of the 12 components including population density, economic activity, traditional markets, modern markets, transportation infrastructure, sanitation, communications, housing, education, diversity, health infrastructure, and social services.

### 2.3. Statistical Analysis

**Figure 1:**
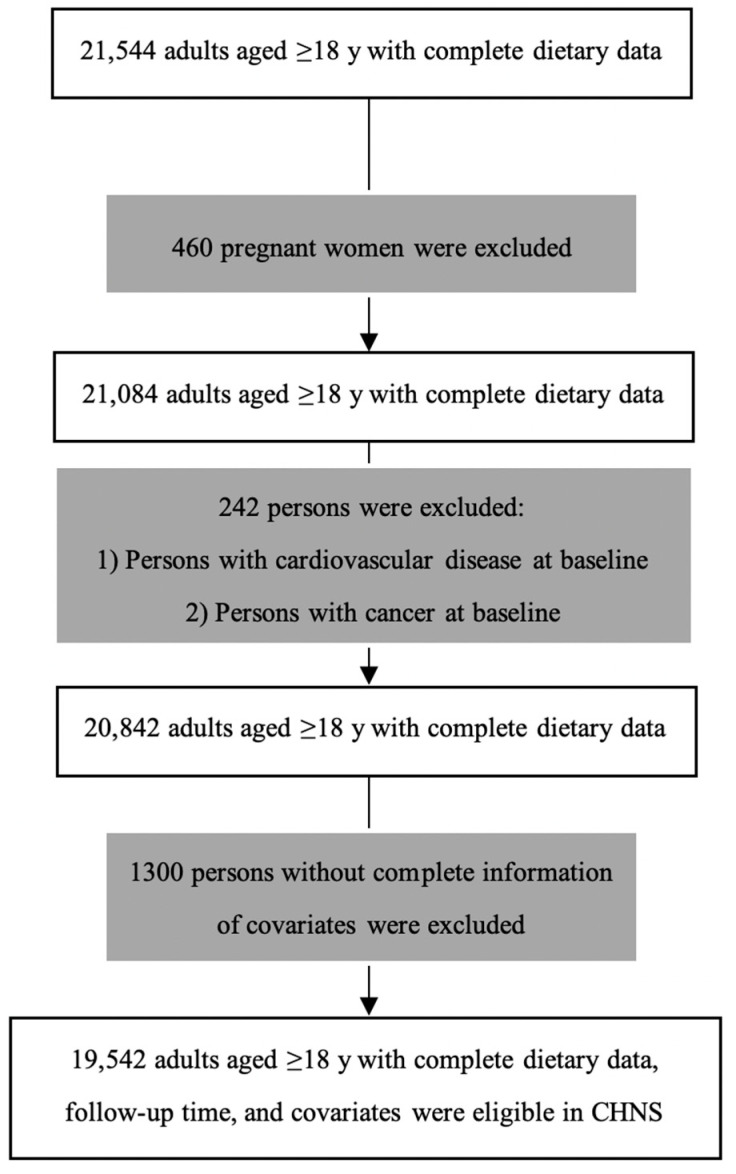
Flow chart of participants in China Health and Nutrition Survey (CHNS).

Complete case analysis was conducted on 19,542 participants. The flow chart of the sample included is shown in Figure 1. FV intake was categorized into quintiles based on their frequency distribution. Descriptive analysis for baseline characteristics was conducted using mean ± SD for continuous variables and % for categorical variables in the overall sample and according to FV intake. Crude and adjusted hazard ratios (HRs) with 95% confidence intervals (CIs) were estimated for all quintile categories of FV intake (combined FV, total fruit, total vegetables) using Cox proportional hazards regression models, with the first quintile used as a reference category. We used a continuous variable calculated from the respective midpoints of the quintiles in the Cox proportional hazard models to test the statistical significance for trends (*p* for trends) in the associations across increasing quintiles of fruit and vegetable intake with mortality. Three models were applied. Model 1 was a crude model; Model 2 was adjusted for age and sex; Model 3 used the complete sample with covariates adjusted for age, sex, education level, income level, marital status, residence, smoking status, alcohol drinking, physical activity categories, body mass index categories, and urban index. Analyses of vegetable intake were adjusted for fruit intake and vice versa. All analyses were conducted in R software 3.5.3 (https://www.r-project.org/) and a two-sided *p* < 0.05 was considered statistically significant.

## 3. Results

### 3.1. Participants’ Characteristics

Among the final sample of 19,542 participants (followed up for a median of 14 years), 1409 participants died. The mean age (SD) of participants at baseline was 41.0 (15.4) years, more than half (53.9%) were women, 72.8% of them completed primary or junior high school, most (84.0%) were in a married relationship, 57.9% lived in urban areas, and the mean intake of calories of the sample was 2183.09 (1190.61) kcal/day. The mean intakes (SD) for fruit, vegetables, and both FV, were respectively: 42.93 (82.14), 311.14 (140.13), and 354.07 (161.63) g/day. Baseline characteristics of study participants categorized by quintiles of FV intake were presented in Table 1. Compared with participants with lower intakes of FV, those who consumed higher amounts were more likely to be younger, women, in a married relationship, and living in rural areas. Such participants were also more likely to be non-smokers, non-alcohol consumers, non-obese, and live in a community which had a higher urban index.

### 3.2. FV Intake and All-Cause Mortality

Table 2 shows the HRs for all-cause mortality according to different quintiles of FV intake. In crude models, increased separate vegetable and fruit intakes as well as combined FV intake compared with the lowest quintile of intake was significantly associated with decreased all-cause mortality (*p* for trend <0.001). In the minimally adjusted (model 2) and fully adjusted models (model 3), the increasing intake of vegetable was not associated with a decreased all-cause mortality (*p* for trend in model 2 = 0.311, *p* for trend in model 3 = 0.234, respectively). However, increased separate fruit intake and combined FV intake measured by quintiles were associated with a significant decreasing trend in all-cause mortality both in minimally adjusted and fully adjusted models (*p* for trend <0.001). In the fully adjusted model, vegetable intake between 327 and 408 g/day was most inversely associated with all-cause mortality (HR = 0.63, 95% CI: 0.53–0.76); intake of fruits in the fifth quintile was most inversely associated with all-cause mortality, at more than 126 g/day (HR = 0.24, 95% CI: 0.15–0.40). Increasing FV intake was significantly inversely associated with all-cause mortality in the fully adjusted model and manifested the strongest protection in the fourth quintile intake (367–461 g/day) compared to the first quintile intake (HR = 0.59; 95% CI: 0.49–0.70).

**Figure 2:**
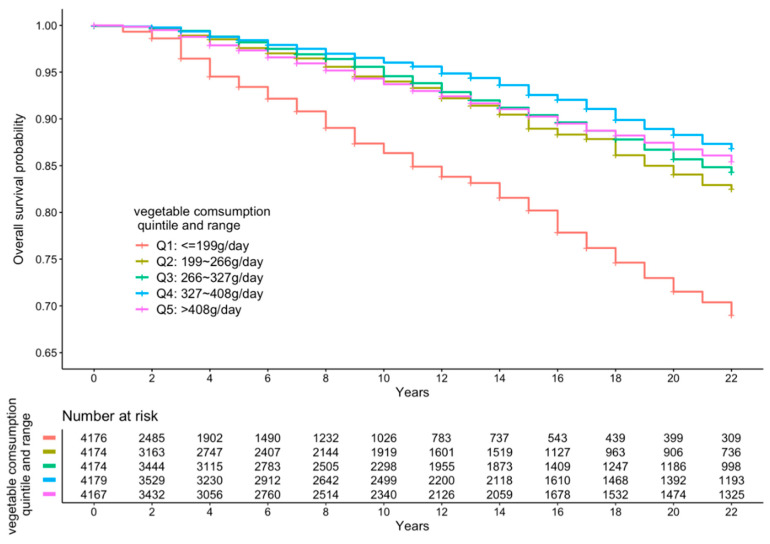
Kaplan–Meier curve of all-cause mortality events according to quintiles of vegetable intake.

**Figure 3:**
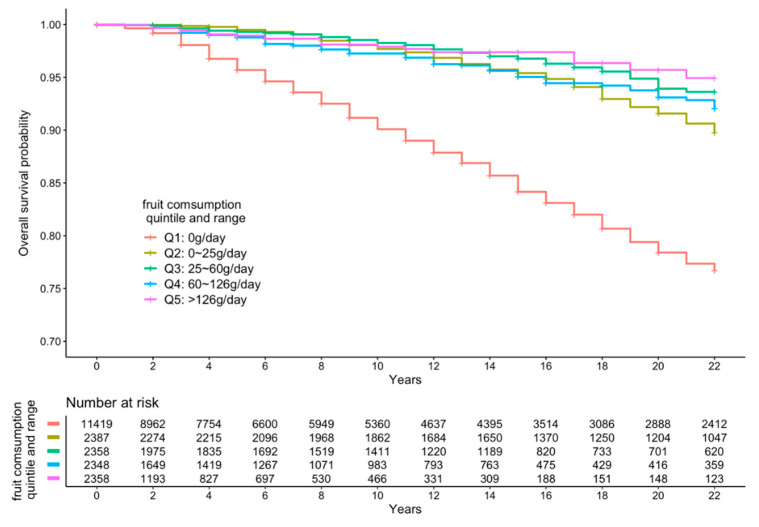
Kaplan–Meier curve of all-cause mortality events according to quintiles of fruit intake.

**Figure 4:**
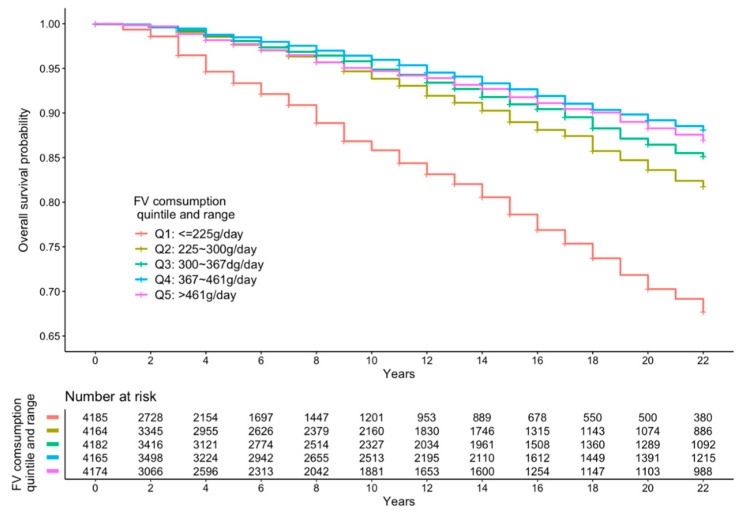
Kaplan–Meier curve of all-cause mortality events according to quintiles of FV intake.

Figure 2, Figure 3 and Figure 4 present the cumulative survival curves for subjects who consumed either vegetables, fruit, or both. For fruit intake higher quintiles were accompanied by increased overall survival probability. In accordance with the results of hazard ratios of vegetable intake and combined FV intake calculated with fully adjusted model, subjects whose vegetable intake was in the fourth quintile showed the highest overall survival rate over a long time period compared with those in the other quintiles, and this result was consistent when subjects consumed vegetable and fruit together. We compared the survival curve of Q2, Q3, Q4, and Q5 with Q1 and found all the *p*-values from the Kaplan–Meier analyses were significant.

## 4. Discussion

To our knowledge, this is the first study, using a nationwide and all-adult sample in China, to explore the association of FV intake, both separately and combined, with all-cause mortality. The findings from our study have the potential to inform policy making and public health interventions on FV intake in China. In the present study, negative associations on mortality risk were observed, starting with the second quintile of intake (199 to ≤266 g/day of vegetables, 0 to ≤25 g/day of fruit and 225 to ≤300 g/day of fruit and vegetables).

Separate intake of vegetables or fruit was associated with reduced mortality from all-causes in all models. If participants consumed vegetables only, with the fully adjusted model, the greatest risk reduction effect of vegetables from all-cause mortality was shown in the amount of the fourth quintile (HR = 0.63, 95% CI: 0.53–0.76), which was about 327–408 g/day. When daily intake of vegetables by individual went beyond 408 g, the protection effect was attenuated by 27% (HR = 0.80, 95%CI: 0.67–0.96). The effect of single vegetable intake appeared to reach the threshold in the fourth quintile (327–408 g/day), beyond which there was no further reduction in mortality. This finding was also observed in combined FV intake; when people consumed fruit and vegetable together, it seemed that the lowest hazard ratio was in the fourth quintile (367–461 g/day) rather than the fifth quintile. These findings are in agreement with a meta-analysis conducted by Wang and his colleagues [17]. They observed a dose–response relationship between fruit and vegetable intake and mortality up to a threshold of three servings/day for vegetables, and five servings/day for fruit and vegetables combined. There was no further reduction in mortality risk beyond these thresholds. Nguyen and his colleagues only observed a threshold of 3 to ≤5 servings for vegetables but not for fruit and vegetables combined [15]. This is plausible because more intake of vegetables does not necessarily mean there are corresponding health benefits. The heavy metals in vegetables which often aggregate in the edible part is of growing concern in China due to the contaminated soils and irrigation water [18]. Pesticide residue in vegetables is also a problem in China which was reported by Xu and her colleagues, showing that ~40% of collected samples tested positive for different kinds of pesticides and 6.1% exceeded the Chinese residue limits [19]. Long term exposure to vegetables with heavy metals and pesticide residues is extremely hazardous to human health [20,21]. High intake of vegetables increases the risk of exposure to these harmful substances which may counteract the benefits of vegetables.

Most participants in this study did not adhere to the amount of daily fruit intake recommended by Chinese Dietary Guidelines (200–350 g/day). The fourth quintile of fruit intake in this group of participants was only from 60 to 126 g/day, and more than 10,000 participants did not even take any fruit every day. The greatest mortality reduction of fruit was in the fifth quintile of intake, with hazard ratio equivalent to 0.24 compared to the first quintile (95% CI: 0.15–0.40). The protective effect of fruit seems to be superior to vegetables or FV combined. This may be due to the fact that more than 10,000 participants do not take any fruit; hence, this negative association with death is obvious once fruit is consumed and appears to be strong at maximum intake of fruit. The message to promote fruit intake in China still needs to be reinforced. At the same time, hazard ratios of fruit intake tended to decrease slightly with more intake of fruit (*p* for trend <0.001). A linear trend across the categories of fruit intake defined by five different quintiles is easily found which suggests that more fruit is advantageous to people’s health.

Intake of FV combined was inversely associated with all-cause mortality in this cohort of Chinese adults in the crude model. After adjustment for age and sex, the protective effect of FV combined was attenuated by about 31% with HR changing from 0.34 (95%CI: 0.28–0.40) to 0.65 (95%CI: 0.55–0.78). After further adding socio-economic, lifestyle, and health-related factors, the association was still consistent and statistically significant, while the protective effect of FV combined decreased by approximately 5% (HR changed from 0.65 to 0.70). It is worth noting that hazard ratios tended to decrease gradually accompanied with more intake of FV with *p* for trend <0.001. Obviously, there is a linear trend across the categories of FV intake defined by five different quintiles which is similar to fruit intake, as mentioned above. The results of the association analysis will also enable more evidence-based design of dietary policy interventions. This research could thus provide insights for policy makers and raise nutrition literacy awareness among the general population. WHO advocated eating at least 400 g of fruit and vegetables per day, while the third quintile of FV intake was even below 367 g/day which means that more than 60% participants in this cohort did not meet WHO standards. More efforts at promoting FV intake—particularly fruit intake—are needed as only a small proportion of Chinese adults currently meet Chinese and WHO recommendations for FV intake.

Findings from the present study are consistent with those from previous prospective cohort studies which have mostly found a significant inverse relationship between FV intake and all-cause mortality [16,22,23,24,25,26,27,28]. A meta-analysis of global prospective cohort studies showed that pooled HRs of all-cause mortality were 0.95 (95% CI: 0.92, 0.98; *p* = 0.001) for an increment of one serving a day of FV, 0.94 (95% CI: 0.90, 0.98; *p* = 0.002) for fruit, and 0.95 (95% CI: 0.92, 0.99; *p* = 0.006) for vegetables [17]. In our study, the risk reduction effects of FV combined intake and individual fruit and vegetable intake levels were also observed as in the aforementioned study but were slightly stronger. In our study, the negative association with death of consuming both fruit and vegetables was slightly stronger than for separate intake of vegetables. The higher negative association of combined FV intake may be partly due to the fruit intake. Because large number of people in this cohort consume no fruit, the risk reduction in mortality due to fruit is comparatively obvious and strong even if people eat just a little more fruit. However, another recent meta-analysis showed reductions in risk of cardiovascular disease and all-cause mortality in those with a combined FV intake reaching up to 800 g/day, whereas no reductions in risk of cancer were observed for those with intakes above 600 g/day [10]. These disparities in findings can be attributed to some factors that varied with studies of different designs, including measures of fruit and vegetable intake, covariates adjustment, follow-up time, and participants’ characteristics.

The main strengths of this study include large nationwide population sampling, which was conducted in municipalities with a range of substantially different geographies, economic development, public resources, and health indicators. This study was prospective, which minimizes bias and provides stronger evidence for causality. In addition, we used a cumulative intake of FV over the cohort period, which may reflect the long-term habitual patterns of dietary behavior. Finally, this study has used a 3 day 24-h recall as a detailed dietary method to reduce measurement error, which provided precise and accurate average values for calorie intake and for foods that are consumed every day such as vegetables, but this is not precise on the individual level regarding foods that are consumed only occasionally such as fruit in this cohort. Usually, food frequency questionnaires provide good differentiation between individuals with high and low consumption of infrequently consumed food, but the averages tend to be less accurate. In our context, we were able to find a difference between the vegetable data and the fruit data because we applied the 3 day 24-h diet data collection method. The higher accuracy of food diary method is a strength in our study.

The study also had several limitations. One limitation is that we did not have information on specific causes of death beyond all-cause mortality, which might be informative to test the association between FV intake and cause-specific mortality in the nationwide population. In addition, there may be some measured and unmeasured covariates that were not included in the analysis. Furthermore, there are many cooking methods and different types of FV; for example, raw vegetables are consumed more in western settings while cooked vegetables are consumed in eastern settings, which may have different implications for health and total mortality. These aspects were not reported on in this study. Finally, the different diet intakes may be indicators of different lifestyles, rather than reflecting effects of specific foods on health, so it is important for future studies to assess the different correlations with age, rural/urban, socio economic groups, and so on. Future studies could also consider including more detailed information of FV intake and linking to cause-specific mortalities.

## 5. Conclusions

Overall, FV intake was associated with a reduced risk of total mortality for Chinese adult populations in this large Chinese cohort. High intake of fruit has a stronger negative association with mortality than differences in intake of vegetables. Further studies examining the effects of FV intake on cause-specific mortality, such as cardiovascular disease mortality, specific cancer, and other diseases are warranted. Our findings support recommendations to increase the intake of vegetables and fruit to promote overall longevity.

## Figures and Tables

**Table 1:** Baseline information by categories of fruit and vegetable (FV) intake in the CHNS Study.

Variable	Overall	Quintiles of Fruit Intake					Quintiles of Vegetable Intake					Quintiles of Combined FV Intake				
		Q1	Q2	Q3	Q4	Q5	Q1	Q2	Q3	Q4	Q5	Q1	Q2	Q3	Q4	Q5
Number of subjects	19,542	10,444	2332	2251	2245	2270	3840	3891	3924	3957	3930	3800	3885	3934	3944	3979
Age (mean (SD))	41.04 (15.37)	40.87 (16.03)	40.15 (14.00)	40.15 (14.56)	41.61 (14.60)	43.09 (14.94)	44.23 (17.27)	41.79 (15.61)	40.68 (14.76)	39.39 (14.29)	39.22 (14.21)	44.00 (17.46)	41.69 (15.66)	40.06 (14.67)	39.35 (13.93)	40.25 (14.52)
Age group (%)																
18–24	2895 (14.8)	1815 (17.4)	283 (12.1)	300 (13.3)	252 (11.2)	245 (10.8)	546 (14.2)	529 (13.6)	556 (14.2)	612 (15.5)	652 (16.6)	579 (15.2)	548 (14.1)	578 (14.7)	574 (14.6)	616 (15.5)
25–34	5073 (26.0)	2595 (24.8)	662 (28.4)	657 (29.2)	603 (26.9)	556 (24.5)	857 (22.3)	1015 (26.1)	1033 (26.3)	1135 (28.7)	1033 (26.3)	846 (22.3)	984 (25.3)	1111 (28.2)	1125 (28.5)	1007 (25.3)
35–44	4182 (21.4)	2104 (20.1)	590 (25.3)	504 (22.4)	528 (23.5)	456 (20.1)	650 (16.9)	789 (20.3)	908 (23.1)	890 (22.5)	945 (24.0)	637 (16.8)	816 (21.0)	868 (22.1)	945 (24.0)	916 (23.0)
45–54	3077 (15.7)	1525 (14.6)	380 (16.3)	365 (16.2)	367 (16.3)	440 (19.4)	608 (15.8)	592 (15.2)	619 (15.8)	611 (15.4)	647 (16.5)	551 (14.5)	587 (15.1)	619 (15.7)	646 (16.4)	674 (16.9)
55–64	2675 (13.7)	1420 (13.6)	274 (11.7)	284 (12.6)	319 (14.2)	378 (16.7)	620 (16.1)	612 (15.7)	534 (13.6)	474 (12.0)	435 (11.1)	625 (16.4)	590 (15.2)	502 (12.8)	442 (11.2)	516 (13.0)
65+	1640 (8.4)	985 (9.4)	143 (6.1)	141 (6.3)	176 (7.8)	195 (8.6)	559 (14.6)	354 (9.1)	274 (7.0)	235 (5.9)	218 (5.5)	562 (14.8)	360 (9.3)	256 (6.5)	212 (5.4)	250 (6.3)
Sex = female (%)	10,532 (53.9)	5353 (51.3)	1261 (54.1)	1251 (55.6)	1288 (57.4)	1379 (60.7)	2296 (59.8)	2174 (55.9)	2183 (55.6)	2057 (52.0)	1822 (46.4)	2155 (56.7)	2188 (56.3)	2141 (54.4)	2076 (52.6)	1972 (49.6)
Body mass index (mean (SD))	23.09 (3.42)	22.78 (3.25)	22.96 (4.18)	23.42 (3.23)	23.62 (3.34)	23.82 (3.38)	23.35 (3.69)	23.05 (3.21)	23.05 (3.21)	23.06 (3.13)	22.86 (3.80)	23.12 (3.57)	23.03 (3.23)	23.06 (3.16)	23.06 (3.16)	23.17 (3.84)
BMI category (%)																
underweight	904 (4.6)	599 (5.7)	93 (4.0)	65 (2.9)	73 (3.3)	74 (3.3)	225 (5.9)	153 (3.9)	169 (4.3)	168 (4.2)	189 (4.8)	236 (6.2)	177 (4.6)	151 (3.8)	167 (4.2)	173 (4.3)
normal weight	11,886 (60.8)	6642 (63.6)	1491 (63.9)	1333 (59.2)	1223 (54.5)	1197 (52.7)	2162 (56.3)	2349 (60.4)	2400 (61.2)	2419 (61.1)	2556 (65.0)	2213 (58.2)	2383 (61.3)	2450 (62.3)	2428 (61.6)	2412 (60.6)
overweight	5330 (27.3)	2546 (24.4)	614 (26.3)	672 (29.9)	732 (32.6)	766 (33.7)	1094 (28.5)	1095 (28.1)	1079 (27.5)	1119 (28.3)	943 (24.0)	1039 (27.3)	1052 (27.1)	1056 (26.8)	1076 (27.3)	1107 (27.8)
obese	1422 (7.3)	657 (6.3)	134 (5.7)	181 (8.0)	217 (9.7)	233 (10.3)	359 (9.3)	294 (7.6)	276 (7.0)	251 (6.3)	242 (6.2)	312 (8.2)	273 (7.0)	277 (7.0)	273 (6.9)	287 (7.2)
Education (%)																
Primary or junior high school	14,235 (72.8)	8330 (79.8)	1929 (82.7)	1598 (71.0)	1318 (58.7)	1060 (46.7)	2429 (63.3)	2718 (69.9)	2957 (75.4)	3001 (75.8)	3130 (79.6)	2661 (70.0)	2907 (74.8)	2962 (75.3)	2934 (74.4)	2771 (69.6)
High school or equal	3823 (19.6)	1656 (15.9)	340 (14.6)	505 (22.4)	612 (27.3)	710 (31.3)	904 (23.5)	842 (21.6)	716 (18.2)	749 (18.9)	612 (15.6)	782 (20.6)	727 (18.7)	730 (18.6)	769 (19.5)	815 (20.5)
College and university	1484 (7.6)	458 (4.4)	63 (2.7)	148 (6.6)	315 (14.0)	500 (22.0)	507 (13.2)	331 (8.5)	251 (6.4)	207 (5.2)	188 (4.8)	357 (9.4)	251 (6.5)	242 (6.2)	241 (6.1)	393 (9.9)
Individual average income ^†^																
Q1	4884 (25.0)	3290 (31.5)	796 (34.1)	454 (20.2)	217 (9.7)	127 (5.6)	570 (14.8)	819 (21.0)	973 (24.8)	1149 (29.0)	1373 (34.9)	730 (19.2)	971 (25.0)	1048 (26.6)	1128 (28.6)	1007 (25.3)
Q2	4901 (25.1)	2805 (26.9)	789 (33.8)	617 (27.4)	466 (20.8)	224 (9.9)	797 (20.8)	1006 (25.9)	1086 (27.7)	1100 (27.8)	912 (23.2)	932 (24.5)	1078 (27.7)	1109 (28.2)	1027 (26.0)	755 (19.0)
Q3	4757 (24.3)	2359 (22.6)	549 (23.5)	685 (30.4)	666 (29.7)	498 (21.9)	866 (22.6)	1032 (26.5)	1085 (27.7)	959 (24.2)	815 (20.7)	901 (23.7)	981 (25.3)	1003 (25.5)	1003 (25.4)	869 (21.8)
Q4	5000 (25.6)	1990 (19.1)	198 (8.5)	495 (22.0)	896 (39.9)	1421 (62.6)	1607 (41.8)	1034 (26.6)	780 (19.9)	749 (18.9)	830 (21.1)	1237 (32.6)	855 (22.0)	774 (19.7)	786 (19.9)	1348 (33.9)
Marital status = married (%)	16,416 (84.0)	8465 (81.1)	2050 (87.9)	1982 (88.0)	1956 (87.1)	1963 (86.5)	3074 (80.1)	3246 (83.4)	3375 (86.0)	3416 (86.3)	3305 (84.1)	3015 (79.3)	3250 (83.7)	3353 (85.2)	3401 (86.2)	3397 (85.4)
Residence = urban (%)	11,324 (57.9)	6910 (66.2)	1455 (62.4)	1197 (53.2)	986 (43.9)	776 (34.2)	1953 (50.9)	2138 (54.9)	2313 (58.9)	2425 (61.3)	2495 (63.5)	2126 (55.9)	2314 (59.6)	2357 (59.9)	2351 (59.6)	2176 (54.7)
Urban index ^‡^ (mean (SD))	58.59 (22.75)	53.72 (21.97)	51.11 (19.11)	59.86 (21.31)	68.69 (20.44)	77.44 (19.25)	66.95 (21.47)	61.90 (21.64)	58.31 (21.63)	54.61 (21.97)	51.44 (23.66)	63.00 (21.55)	58.56 (21.42)	56.95 (21.93)	55.30 (22.40)	59.30 (25.39)
Smoking status																
Current	2920 (14.9)	1537 (14.7)	201 (8.6)	337 (15.0)	405 (18.0)	440 (19.4)	684 (17.8)	610 (15.7)	507 (12.9)	537 (13.6)	582 (14.8)	674 (17.7)	543 (14.0)	517 (13.1)	515 (13.1)	671 (16.9)
Not current (never, past)	7565 (38.7)	3433 (32.9)	491 (21.1)	887 (39.4)	1200 (53.5)	1554 (68.5)	1966 (51.2)	1564 (40.2)	1454 (37.1)	1364 (34.5)	1217 (31.0)	1672 (44.0)	1406 (36.2)	1392 (35.4)	1404 (35.6)	1691 (42.5)
Not answered	9057 (46.3)	5474 (52.4)	1640 (70.3)	1027 (45.6)	640 (28.5)	276 (12.2)	1190 (31.0)	1717 (44.1)	1963 (50.0)	2056 (52.0)	2131 (54.2)	1454 (38.3)	1936 (49.8)	2025 (51.5)	2025 (51.3)	1617 (40.6)
Alcohol drinking																
Current	3603 (18.4)	1735 (16.6)	232 (9.9)	432 (19.2)	549 (24.5)	655 (28.9)	871 (22.7)	731 (18.8)	664 (16.9)	643 (16.2)	694 (17.7)	785 (20.7)	659 (17.0)	648 (16.5)	648 (16.4)	863 (21.7)
Not current (never, past)	6838 (35.0)	3211 (30.7)	456 (19.6)	788 (35.0)	1052 (46.9)	1331 (58.6)	1778 (46.3)	1429 (36.7)	1291 (32.9)	1249 (31.6)	1091 (27.8)	1555 (40.9)	1284 (33.1)	1251 (31.8)	1260 (31.9)	1488 (37.4)
Not answered	9101 (46.6)	5498 (52.6)	1644 (70.5)	1031 (45.8)	644 (28.7)	284 (12.5)	1191 (31.0)	1731 (44.5)	1969 (50.2)	2065 (52.2)	2145 (54.6)	1460 (38.4)	1942 (50.0)	2035 (51.7)	2036 (51.6)	1628 (40.9)
Physical activity																
Light	10,273 (52.6)	4623 (44.3)	1015 (43.5)	1307 (58.1)	1563 (69.6)	1765 (77.8)	2701 (70.3)	2374 (61.0)	2067 (52.7)	1731 (43.7)	1400 (35.6)	2454 (64.6)	2166 (55.8)	1955 (49.7)	1821 (46.2)	1877 (47.2)
Moderate	3537 (18.1)	2038 (19.5)	519 (22.3)	395 (17.5)	325 (14.5)	260 (11.5)	577 (15.0)	716 (18.4)	760 (19.4)	788 (19.9)	696 (17.7)	652 (17.2)	762 (19.6)	766 (19.5)	713 (18.1)	644 (16.2)
Heavy	5732 (29.3)	3783 (36.2)	798 (34.2)	549 (24.4)	357 (15.9)	245 (10.8)	562 (14.6)	801 (20.6)	1097 (28.0)	1438 (36.3)	1834 (46.7)	694 (18.3)	957 (24.6)	1213 (30.8)	1410 (35.8)	1458 (36.6)
Calorie intake (kcal/day) (mean (SD))	2183.09 (1190.61)	2228.19 (1287.02)	2229.97 (546.66)	2129.02 (582.43)	2071.18 (1356.15)	2091.75 (1444.66)	1993.62 (778.84)	2178.29 (502.93)	2238.84 (425.09)	2370.71 (507.87)	2511.44 (1175.61)	1925.57 (1147.12)	2093.93 (616.98)	2187.04 (619.84)	2284.93 (597.35)	2411.24 (2113.77)

^†^ At the exchange rate of Aug 2019, 1 yuan was approximately equal to 0.14 U.S. dollars. ^‡^ Measured at the community level on a 12-component continuous scale ranging from 0–120 with higher values corresponding to higher levels of urbanicity. The quintiles of intake for fruit and vegetables (g/day) were as follows: Vegetables: Q1: <199; Q2: 199 to ≤266; Q3: 266 to ≤327; Q4: 327 to ≤408; Q5: >408. Fruit: Q1: ≤0; Q2: 0 to ≤25; Q3: 25 to ≤60; Q4: 60 to ≤126; Q5: >126. Fruit and vegetable combined: Q1: <225; Q2: 225 to ≤300; Q3: 300 to ≤367; Q4: 367 to ≤461; Q5: >461.

**Table 2:** Hazard ratios and 95 % confidence intervals of all-cause mortality by quintiles of intake for FV (n = 19,542).

	Quintiles ^a^										*P for Trend*
	Q1		Q2		Q3		Q4		Q5		
**Vegetable intake**											
Model 1 (crude)	1	Reference	0.52	(0.44–0.62)	0.46	(0.39–0.54)	0.37	(0.31–0.44)	0.46	(0.39–0.55)	<0.001
Model 2 ^b^ (age, sex adjusted)	1	Reference	0.71	(0.60–0.85)	0.74	(0.62–0.87)	0.66	(0.55–0.79)	0.93	(0.79–1.11)	0.311
Model 3 ^c^ (adjusted)	1	Reference	0.78	(0.65,0.93)	0.75	(0.63,0.89)	0.63	(0.53,0.76)	0.8	(0.67,0.96)	0.234
**Fruit intake**											
Model 1 (crude)	1	Reference	0.35	(0.29–0.41)	0.19	(0.15–0.25)	0.24	(0.18–0.31)	0.13	(0.08–0.21)	<0.001
Model 2 ^b^ (age, sex adjusted)	1	Reference	0.34	(0.29–0.41)	0.23	(0.18–0.30)	0.30	(0.23–0.39)	0.13	(0.08–0.21)	<0.001
Model 3 ^c^ (adjusted)	1	Reference	0.36	(0.30–0.43)	0.28	(0.22–0.36)	0.43	(0.32–0.57)	0.24	(0.15–0.40)	<0.001
**FV intake**											
Model 1 (crude)	1	Reference	0.48	(0.41–0.56)	0.38	(0.32–0.44)	0.29	(0.25–0.35)	0.34	(0.28–0.40)	<0.001
Model 2 ^b^ (age, sex adjusted)	1	Reference	0.68	(0.58–0.8)	0.61	(0.52–0.72)	0.56	(0.47–0.67)	0.65	(0.55–0.78)	<0.001
Model 3 ^c^ (adjusted)	1	Reference	0.71	(0.61–0.84)	0.63	(0.54–0.75)	0.59	(0.49–0.70)	0.70	(0.58–0.85)	<0.001

^a^ The quintiles of intake for fruit and vegetables (g/day) were as follows: Vegetables: Q1: <199; Q2: 199 to ≤266; Q3: 266 to ≤327; Q4: 327 to ≤408; Q5: >408. Fruit: Q1: <0; Q2: 0 to ≤25; Q3: 25 to ≤60; Q4: 60 to ≤126; Q5: >126. Fruit and vegetables combined: Q1: <225; Q2: 225 to ≤300; Q3: 300 to ≤367; Q4: 367 to ≤461; Q5: >461. ^b^ Model 2 was adjusted for age and sex. ^c^ Model 3 was adjusted for age, sex, education level, marital status, individual average income, residence, physical activity categories, smoking status, alcohol drinking, calorie intake, urban index and body mass index categories.

## Data Availability

Publicly available datasets were analyzed in this study. This data can be found here: [https://www.cpc.unc.edu/projects/china].

## References

[1] Dhandevi P., Jeewon R. (2015). Fruit and vegetable intake: Benefits and progress of nutrition education interventions-narrative review article. Iran. J. Public Health.

[2] Li X.-T., Liao W., Yu H.-J., Liu M.-W., Yuan S., Tang B.-W., Yang X.-H., Song Y., Huang Y., Cheng S.-L. (2017). Combined effects of fruit and vegetables intake and physical activity on the risk of metabolic syndrome among Chinese adults. PLoS ONE.

[3] Xiao H.-J., Liang H., Wang J.-B., Huang C.-Y., Wei W.-Q., Boniol M., Qiao Y.-L., Boffetta P. (2011). Attributable causes of cancer in China: Fruit and vegetable. Chin. J. Cancer Res..

[4] Yang Y. Expert Interpretation-New Dietary Guidelines (3) Eat More Fruits and Vegetables, Milk, Soybeans. http://dg.cnsoc.org/article/04/8a2389fd575f695101577a3abfdd02d7.html.

[5] Zhao L., Fang Y., He Y., Yu D., Guo Q., Yu W., Zhao W. (2016). Trends of food intake among Chinese population in 1992–2012. J. Hyg. Res..

[6] Li Y.C., Jiang B., Zhang M., Huang Z.J., Qian DE N.G., Zhou M.G., Wang L.M. (2017). Vegetable and fruit intake among Chinese adults and associated factors: A nationally representative study of 170,847 adults. Biomed. Environ. Sci..

[7] Kaur H., Aeri B.T. (2019). Protective Impact of Fruits and Vegetable Intake on Cardiovascular Risk Factors-A Review. J. Clin. Diagn. Res..

[8] Yahia E.M. (2010). The Contribution of Fruit and Vegetable Intake to Human Health. Fruit and Vegetable Phytochemicals.

[9] Du H., Li L., Bennett D., Yang L., Guo Y., Key T.J., Chen J. (2017). Fresh fruit intake and all-cause and cause-specific mortality: Findings from the China Kadoorie Biobank. Int. J. Epidemiol..

[10] Aune D., Giovannucci E., Boffetta P., Fadnes L.T., Keum N., Norat T., Greenwood D.C., Riboli E., Vatten L.J., Tonstad S. (2017). Fruit and vegetable intake and the risk of cardiovascular disease, total cancer and all-cause mortality—A systematic review and dose-response meta-analysis of prospective studies. Int. J. Epidemiol..

[11] Du H., Li L., Bennett D., Guo Y., Key T.J., Bian Z., Chen J. (2016). Fresh Fruit Intake and Major Cardiovascular Disease in China. N. Eng. J. Med..

[12] Nechuta S.J., Shu X.-O., Li H.-L., Yang G., Xiang Y.-B., Cai H., Chow W.-H., Ji B., Zhang X., Wen W. (2010). Combined Impact of Lifestyle-Related Factors on Total and Cause-Specific Mortality among Chinese Women: Prospective Cohort Study. PLoS Med..

[13] Wang Y., Yan R., Yin L., Chen H., Li W. (2018). A8560 Fruit, vegetable, and legume intake and mortality risk among China adults. J. Hypertens..

[14] Popkin B., Du S., Zhai F., Zhang B. (2009). Cohort Profile: The China Health and Nutrition Survey--monitoring and understanding socio-economic and health change in China, 1989–2011. Int. J. Epidemiol..

[15] Nguyen B., Bauman A., Gale J., Banks E., Kritharides L., Ding D. (2016). Fruit and vegetable intake and all-cause mortality: Evidence from a large Australian cohort study. Int. J. Behav. Nutr. Phys. Act..

[16] Batis C., Sotres-Alvarez D., Gordon-Larsen P., Mendez M.A., Adair L., Popkin B. (2014). Longitudinal analysis of dietary patterns in Chinese adults from 1991 to 2009. Br. J. Nutr..

[17] Wang X., Ouyang Y., Liu J., Zhu M., Zhao G., Bao W., Hu F.B. (2014). Fruit and vegetable consumption and mortality from all causes, cardiovascular disease, and cancer: Systematic review and dose-response meta-analysis of prospective cohort studies. BMJ.

[18] Pan X.-D., Wu P.-G., Jiang X.-G. (2016). Levels and potential health risk of heavy metals in marketed vegetables in Zhejiang, China. Sci. Rep..

[19] Xu X., Li L., Huang X., Lin H., Liu G., Xu D., Jiang J. (2018). Survey of Four Groups of Cumulative Pesticide Residues in 12 Vegetables in 15 Provinces in China. J. Food Prot..

[20] Engwa G.A., Ferdinand P.U., Nwalo F.N., Unachukwu M.N. (2019). Mechanism and Health Effects of Heavy Metal Toxicity in Humans. Poisoning in the Modern World–New Tricks for an Old Dog?.

[21] Kim K.-H., Kabir E., Jahan S.A. (2017). Exposure to pesticides and the associated human health effects. Sci. Total. Environ..

[22] Okuda N., Miura K., Okayama A., Okamura T., Abbott R.D., Nishi N., Fujiyoshi A., Kita Y., Nakamura Y., Miyagawa N. (2015). Fruit and vegetable intake and mortality from cardiovascular disease in Japan: A 24-year follow-up of the NIPPON DATA80 Study. Eur. J. Clin. Nutr..

[23] Choi Y., Lee J.E., Bae J.-M., Li Z.-M., Kim D.-H., Lee M.-S., Ahn Y.-O., Shin M.-H. (2015). Vegetable Intake, but Not Fruit Intake, Is Associated with a Reduction in the Risk of Cancer Incidence and Mortality in Middle-Aged Korean Men. J. Nutr..

[24] Oyebode O., Gordondseagu V.L.Z., Walker A., Mindell J.S. (2014). Fruit and vegetable consumption and all-cause, cancer and CVD mortality: Analysis of Health Survey for England data. J. Epidemiol. Community Health.

[25] Leenders M., Sluijs I., Ros M.M., Boshuizen H.C., Siersema P.D., Ferrari P., Clavel-Chapelon F. (2013). Fruit and Vegetable Intake and Mortality European Prospective Investigation into Cancer and Nutrition. Am. J. Epidemiol..

[26] Bellavia A., Larsson S.C., Bottai M., Wolk A., Orsini N. (2013). Fruit and vegetable intake and all-cause mortality: A dose-response analysis. Am. J. Clin. Nutr..

[27] Zhang X., Shu X.O., Xiang Y.B., Yang G., Li H., Gao J., Zheng W. (2011). Cruciferous vegetable intake is associated with a reduced risk of total and cardiovascular disease mortality. Am. J. Clin. Nutr..

[28] Nakamura K., Nagata C., Oba S., Takatsuka N., Shimizu H. (2008). Fruit and Vegetable Intake and Mortality from Cardiovascular Disease Are Inversely Associated in Japanese Women but Not in Men. J. Nutr..

